# Intrapericardial Thymoma Presented as Pericardial Tamponade with Post-Operative Myasthenia Gravis

**DOI:** 10.3390/medicina58050609

**Published:** 2022-04-27

**Authors:** Yueh-Hsun Tsai, Kai-Hsiung Ko, Hao Yen, Tsai-Wang Huang

**Affiliations:** 1 Department of Surgery, Tri-Service General Hospital, National Defense Medical Center, Taipei 11490, Taiwan; drxelamai@gmail.com; 2 Department of Radiology, National Defense Medical Center, Tri-Service General Hospital, Taipei 11490, Taiwan; m860818@gmail.com; 3 Department of Pathology, National Defense Medical Center, Tri-Service General Hospital, Taipei 11490, Taiwan; kastyplanet@gmail.com; 4 Division of Thoracic Surgery, Department of Surgery, National Defense Medical Center, Tri-Service General Hospital, Taipei 11490, Taiwan

**Keywords:** thymoma, pericardium, ectopic thymoma, myasthenia gravis, pericardial tamponade

## Abstract

Background: Thymoma is an epithelial mass arising from the thymus. Most thymomas are located in the anterior mediastinum. Ectopic intrapericardial thymoma is very unusual; to date, only eight cases of pericardial thymoma have been reported. Among thymoma patients, 20% to 25% are associated with myasthenia gravis. However, postoperative myasthenia gravis occurs in less than 1% of cases. Here, we share a rare case of ectopic intrapericardial thymoma that developed postoperative myasthenia gravis six months after surgery. Case presentation: A 66-year-old woman visited the outpatient department due to productive cough and chest pain. Chest radiography showed increased soft tissue opacity over the mediastinum. A soft tissue mass in the pericardium and a ground glass nodule in right upper lung were noted using chest computed tomography. The diagnosis of thymoma, type B2, pT3N0M0, and stage IIIA and synchronous adenocarcinoma in situ of the right upper lung was confirmed after surgical removal. Six months later, the patient developed postoperative myasthenia gravis. Conclusions: Thymoma is rarely considered a differential diagnosis in pericardial tumors. Surgical removal with adjuvant radiation therapy should be performed considering the malignancy potential of thymomas and cardiac complications. In patients without myasthenia gravis, a small chance of postoperative myasthenia gravis remains. Patients should be carefully monitored for myasthenia gravis after surgery.

## 1. Introduction

Thymoma is a rare epithelial mass arising from the thymus. More than 96% of thymomas are discovered in the anterior mediastinum. Ectopic thymomas are found most frequently in the cervical region, followed by the lung, pleura and thyroid [1]. Ectopic pericardial thymoma is very unusual; according to a literature review, eight cases of pericardial thymoma have been reported [2,3,4,5,6,7,8]. Among thymoma patients, 20% to 25% are associated with myasthenia gravis [9]. To date, only one case of pericardial thymoma with myasthenia gravis (MG) has been documented [5]. Here, we present a rare case of pericardial thymoma with myasthenia gravis.

## 2. Detailed Case Description

**Figure 1:**
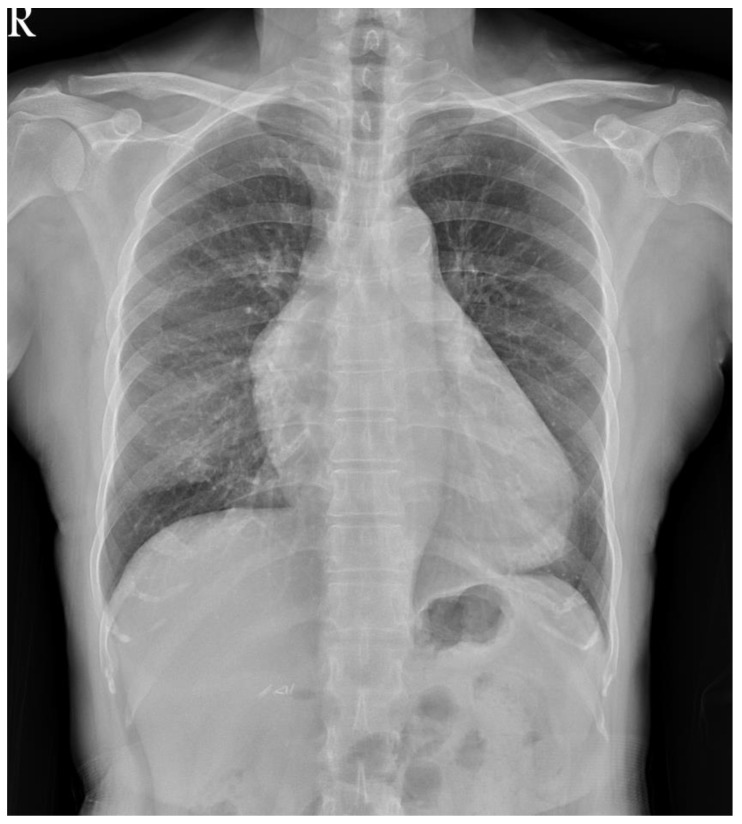
Chest radiography revealed increased soft tissue opacity over the mediastinum.

**Figure 2:**
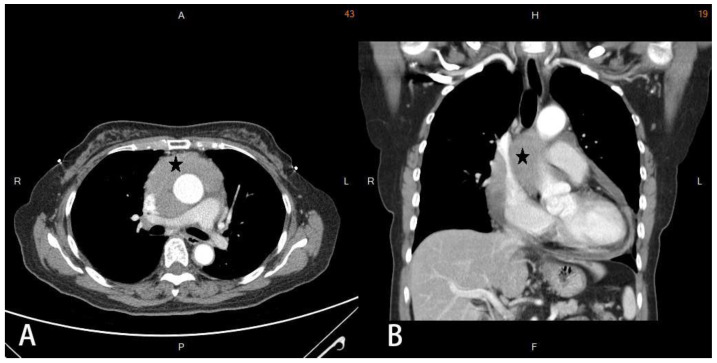
Chest computed tomography with contrast. Axial view (**A**) and coronal view (**B**) of chest computed tomography showing confluent soft tissue masses (asterisk) in the intrapericardial space, with an encasement of the aorta and superior vena cava as well as compression of the right pulmonary artery.

**Figure 3:**
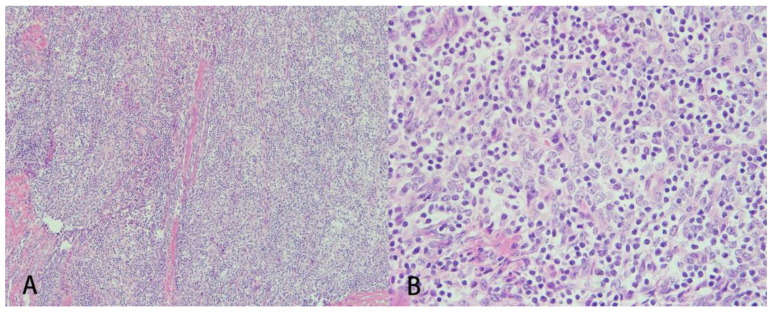
Microscopic findings of the tumor. The section (**A**) shows a hypercellular background composed of round to ovoid epithelioid tumor cells with (**B**) hyperchromatic nuclei intermixed with mature lymphocytes (×400 magnification).

A 66-year-old woman with a history of adrenal insufficiency under regular medication control suffered from a chronic cough for 2 years. She visited the outpatient department due to a worsened productive cough and chest pain. Chest radiography showed increased soft tissue opacity over the mediastinum (Figure 1). Contrast-enhanced computed tomography (CT) of the chest revealed confluent soft tissue masses in the intrapericardial space with the encasement of the aorta and superior vena cava as well as the compression of the right pulmonary artery (Figure 2) and a ground glass nodule of about 0.7 cm in the right upper lung suspected to be adenocarcinoma or atypical adenomatous hyperplasia. Echocardiography revealed a soft tissue mass in the pericardial space with pericardial effusion near the right ventricle. The ejection fraction was estimated to be 53%. A whole abdomen sonography was performed for tumor staging, which reported no significant abnormality other than moderate fatty liver. Physical examinations were unremarkable. Laboratory examinations were within the normal ranges. Median sternotomy with mediastinal tumor resection combined with video assisted thoracic surgery with the right upper lobe of the lung and mediastinal lymph node dissection were performed under general anesthesia. After opening the pericardium, a large pericardial tumor with superior vena cava, aorta, pulmonary artery, and posterior mediastinum muscle involvement was noted. Pericardiocentesis was performed to obtain 120 mL of pericardial fluid. The 11 cm × 8 cm × 1.5 cm tumor was brown in color with a solid consistency. After a debulking resection, the specimen was sent for pathological evaluation. The pathology report revealed that the tumor microscopically consisted of neoplastic epithelioid cells with hyperchromatism intermixed with scattered mature lymphocytes (Figure 3). The immunohistochemical staining was positive for cytokeratin and negative for CD5 and ERG. The finding was compatible with predominant B2 thymoma. The right upper lung tumor was confirmed as adenocarcinoma in situ. The diagnosis of thymoma, type B2, pT3N0M0, and stage IIIA and synchronous adenocarcinoma in situ of the right upper lung was confirmed. The patient was discharged uneventfully two weeks after the surgery, and adjuvant radiotherapy was arranged.

Six months later, the patient complained of diplopia, numbness over the bilateral lower limbs and chest pain. She was referred to the neurology department, where neurological examination revealed paroxysmal diplopia and limb numbness. Myasthenia gravis was confirmed by acetylcholine receptor antibody > 4 nmol/L. The patient’s symptoms resolved after receiving plasma exchange therapy. Her condition was controlled with adjuvant radiation therapy. The patient had regular follow ups with chest CT every six months. The result was normal until 3 years after the surgery. Dry cough and back pain were reported by the patient. A PET-CT revealed a fluorodeoxyglucose-avid nodular lesion at the anterior mediastinum (pericardia space, size: 0.6 cm; standard uptake value max: 3.1), indicating the potential recurrence of thymoma. Left-sided video assisted thoracic surgery with mediastinal tumor resection and lymph node dissection of lymph node 5 and 10 were performed smoothly this time. The pathology confirmed the diagnosis of thymoma, type B2, rpT3N0M0, stage IIIA and Masaoka stage III. The patient was discharged uneventfully two weeks after the surgery. Her condition was stable with a regular follow up every 3 months.

## 3. Discussion

Thymoma is a rare epithelial tumor arising from the thymus that has an incidence of 0.13 per 100,000 persons [10]. Most thymomas are located in the anterior mediastinum, and ectopic thymomas have only been reported in 4% of cases [1]. The formation of ectopic thymoma is considered to arise from remnant thymic tissue during embryogenesis. Normally, the thymus develops from the ventral portion of the third pharyngeal pouch during the sixth week of gestation. During the eighth week, the primordial thymus moves toward its lower poles to form two epithelial bars that fuse in the midline and occupy the final position in the base of the heart. The organ usually vanishes during the final descent. However, if it fails to migrate to the anterior mediastinum, the remnant thymic tissue may turn into ectopic thymic neoplasia [1,6].

The most common sites of ectopic thymomas are the cervical region, lung, pleura and thyroid. Pericardial thymoma is very rare. To our knowledge, only eight cases of pericardial thymoma have been reported [2,3,4,5,6,7,8]. We have summarized the clinical features in Table 1, and include our case. The age varied from 27 to 82 years old, with a mean age of 63.56 years. There were six females and three males. Two cases were autopsies. The mean size of the tumors was 8.3 cm (range 1.5–13 cm). The clinical manifestations included chest pain, cough, dyspnea, orthopnea, dysphonia, and significant body weight loss. Only one case was asymptomatic. According to the World Health Organization classification (WHO), there were two cases of type A, two of type B2, one of type B3 and one of type AB. Three cases were unclassified in the documents. In our review, there were two cases of myasthenia gravis (No. 6 and our case). Statistically, myasthenia gravis occurs in 20% to 25% of thymoma patients [9]. In previous studies, the prevalence of MG was highest in WHO type B2 thymoma patients [11]. This result is consistent with our review, as both cases of type B2 thymoma developed severe myasthenia gravis. In our study, pericardial thymomas were discovered more frequently in females, with a female to male ratio of 2:1, while the distribution was nearly equal among thymoma patients. The age distribution was close to that of anterior mediastinal thymoma, with a mean age of 60 to 70 years [10].

Preoperatively, pericardial thymomas are difficult to distinguish from neoplasms such as lymphoma, angiosarcoma or pleuropulmonary neoplasms. The immunohistochemical features are important clues for differential diagnosis. Thymomas consist of epithelial tumors that adhere to cytokeratins. Markers including p63, p40 and Pax8 are helpful for distinguishing pleuropulmonary neoplasms [1].

The incidence of ectopic thymomas is very rare. The treatment is mainly based on the experience of anterior mediastinal thymomas. Surgical resection is indicated in all patients if the tumor is resectable. Radiation therapy is suggested in patients with unresectable disease or when R0 resection cannot be performed [12]. The indications for radiation therapy in Masaoka stage II disease remain controversial. A recent meta-analysis concluded that postoperative radiation therapy was suggested in Masaoka stages II and III [13].

The onset of MG in thymoma patients generally occurs before thymectomy. However, in our case, the patient developed MG after removal of the thymoma. In previous reports, the onset of postoperative MG appeared in 1% of cases without MG. The mechanism of postoperative MG remains unclear. Some studies suggest that mature T cells are transferred into the peripheral blood before removal, potentially stimulating autoantibody production [14]. According to the study performed by Gamez et al. [15], preoperative intravenous immunoglobulin (IVIg) to prevent myasthenia crisis is not warranted in well-controlled MG patients. It was concluded that IVIg therapy to prevent myasthenia crisis is not necessary in well-controlled myasthenia gravis patients scheduled for surgery. For our case, there was no study suggesting preventive IVIg treatment before thymectomy in non-MG thymoma patients. More clinical studies should be conducted. However, the benefit should be evaluated carefully since MG is only reported to occur in 1% of cases after the removal of a thymoma.

## 4. Conclusions

In conclusion, thymoma is rarely considered a differential diagnosis in pericardial tumors. However, thymomas located in the pericardium can cause severe complications, such as cardiac tamponade or heart failure. Surgical removal with adjuvant radiation therapy should be performed considering the malignancy potential of thymomas and cardiac complications. In patients without MG, a small chance of postoperative MG remains. Patients should be carefully monitored for MG after surgery.

## Figures and Tables

**Table 1:** The characteristics of the patients with pericardial ectopic thymoma.

Case No	Author	Age	Sex	Presentation	Myasthenia Gravis	Tumor Size	WHO Classification	Treatment	Pathology
1	Iliceto	27	F	chest pain	Absent	NA	NA	Surgery + Chemotherapy	epithelial spindle cell
2	Mirra	61	F	weight loss	Absent	4 × 2 × 2 cm	A	NA	Spindle cells, lymphocytes
3	Mirra	82	F	heart failure	Absent	10 × 6 × 4 cm	A	NA	Spindle cells, lymphocytes
4	Deveci	57	M	None	Absent	15 × 10 × 8 mm,	NA	Surgery	rhabdomyomatous thymoma
5	Theodore	62	F	significant	Absent	16 × 13 × 9 cm	NA	Surgery	lympho-epithelial type of thymoma
6	Azoulay	72	F	weight loss	Present	6 × 4 × 3 cm	B2	Surgery	mixed lymphocytic and polygonal epithelial cell type
7	Arai	72	M	dyspnea, dysphonia	Absent	50 mm in diameter	AB	Surgery + radiation therapy	Spindle cells and polygonal epithelial cells
8	Zoroufian	73	M	Heart failure	Absent	13 cm	B3	Surgery	polygonal epithelial cells and lymphocytes
9	Present study	66	F	dyspnea, cough	Present	11 × 8 × 1.5 cm	B2	Surgery + radiation therapy	epithelioid cells mixed with mature lymphocytes

## Data Availability

Not applicable.
